# Quantification of the efficiency of treatment of *Anopheles gambiae *breeding sites with petroleum products by local communities in areas of insecticide resistance in the Republic of Benin

**DOI:** 10.1186/1475-2875-6-56

**Published:** 2007-05-08

**Authors:** Rousseau F Djouaka, Adekunle A Bakare, Honore S Bankole, Julien MC MC Doannio, Hortense Kossou, Martin C Akogbeto

**Affiliations:** 1 Centre de Recherche Entomologique de Cotonou, 06 BP 2604, Republic of Benin; 2 Department of Zoology, Cell Biology and Genetics Unit, University of Ibadan, Nigeria; 3 Ministry of Health, 05BP2099 Cotonou, Republic of Benin; 4 National Institute of Public Health, BPV47 Abidjan, Côte d'Ivoire

## Abstract

**Background:**

The emergence of *Anopheles *populations capable of withstanding lethal doses of insecticides has weakened the efficacy of most insecticide based strategies of vector control and, has highlighted the need for developing new insecticidal molecules or, improving the efficacy of existing insecticides or abandoning those to which resistance has emerged. The use of petroleum products (PP) against mosquito larvae had an immense success during early programmes of malaria control, but these compounds were abandoned and replaced in the 1950s by synthetic insecticides probably because of the high performances given by these new products. In the current context of vector resistance, it is important to elucidate the empirical use of PP by quantifying their efficiencies on resistant strains of *Anopheles*.

**Methods:**

Larvae of *Anopheles *Ladji a local resistant strain were exposed to increasing concentrations of various PP (kerosene, petrol and engine oils) for 24 hours and the lethal activities recorded. The highest concentration (HiC) having no lethal activity (also referred as the NOEL or no effect level) and the lowest concentration (LoC_100_) yielding 100% mortality were rated for each PP on the Ladji strain. Prior to laboratory analysis, KAP studies were conducted in three traditional communities were insecticide resistance is clearly established to confirm the use of PP against mosquitoes.

**Results:**

Laboratory analysis of petrol, kerosene and engine oils, clearly established their lethal activities on resistant strains of *Anopheles *larvae. Contrary to existing references, this research revealed that exposed larvae of *Anopheles *were mostly killed by direct contact toxicity and not by suffocation as indicated in some earlier reports.

**Conclusion:**

This research could serve as scientific basis to backup the empirical utilisation of PP on mosquito larvae and to envisage possibilities of using PP in some traditional settings where *Anopheles *have developed resistance to currently used insecticides.

## Background

Malaria is the deadliest vector-borne disease in the world with 1.5 to 3 million deaths a year. More than 90 % of the deaths associated with this disease are found in Africa and the victims are mainly those with low immune responses such as children [1,2]. In 1992, the WHO set up sustainable strategies against malaria disease, focused on the proper treatment of malaria cases and the use of preventive measures against the vectors. Two main preventive measures are presently used against malaria vectors: indoor residual spraying of insecticides (IRS) and the use of insecticide-treated nets (ITNs). Both methods have been very effective in the control of *Anopheles *mosquito [3-9]. However, with the emergence of populations of *Anopheles *capable of withstanding lethal doses of insecticides, the efficacy of insecticide-based vector control tools is critically affected [10]. The failure of all currently available insecticides has highlighted the importance of research focused on the development of new insecticides or the improvement of existing formulations of insecticides.

Initially used as 'ancestral insecticides', petroleum products (PP), such as petrol, kerosene, engine oil and waste oil, have in the recent past produced spectacular results for larviciding in several communities and, were advocated for vector control by several National Malaria Control Programmes. Their chemical composition appears to confer them immense insecticidal properties which have not yet been investigated thoroughly. Their properties should be properly researched as they could potentially be exploited in areas of resistance. Spreading kerosene on mosquito breeding sites was among the first strategy adopted by early malaria control programmes [11,12]. This traditional vector control method is still in use in communities where the populations are poor and do not have the financial resources to employ conventional insecticides.

The combined sociological and entomological research presented in this paper aims to support the empirical use of PP in areas of resistance by quantifying their lethal activities and their mode of action against *Anopheles *Ladji (the most resistant species of *Anopheles *in Benin). Data generated from this research could serve as a basis to improve the performances of PP as potential alternatives to current synthetic insecticides where resistance has appeared and also to predict the concentrations of PP which could lead to the emergence of pyrethroid resistance.

## Materials and methods

### KAP studies on the empirical utilisation of PP by traditional communities in areas of insecticide resistance

Prior to assessing the larvicidal activities of PP, focus group discussions (FGD), in-depth interviews and questionnaires were conducted in some traditional communities were PP are still used for larval control. The rationale of FGD and interviews was to generate enough information on: i) the empirical use of PP, ii) the mode of application, iii) the periods and eventually iv) the frequencies of utilization of PP.

### Quantification of larvicidal activities of PP on *Anopheles *Ladji

#### Description of the strain of mosquito used in this assay

*Anopheles gambiae *Ladji was selected from the traditional community of Ladji, located about 5–7 km from Cotonou, the economic capital of the Republic of Benin. In this locality, living standards are very low and populations use several traditional methods of mosquito protection including the application of petroleum products to standing water bodies. In these communities, the protection of the environment is of less priority to populations and individuals could continue spraying these products as long as they kill mosquitoes. *A. gambiae *Ladji is "M" form and its level of susceptibility to permethrin and DDT is respectively 65% and 50% mortality rates. The *kdr *mutation, which is one of the mechanisms conferring the resistant status to this strain appears to be homozygous in this mosquito population, with an allelic frequency of one [13].

### Determination of lethal concentrations of PP on larvae of *Anopheles *ladji

Larvae of *Anopheles *Ladji were exposed to four petroleum products, petrol, kerosene, engine oil and waste oil from mechanics, and their larvicidal activities were recorded and quantified. Lethal concentrations of the various petroleum products were determined after treatment of *Anopheles *breeding sites with increasing doses of PP in the laboratory. For each petroleum product, the lowest concentration (LoC_100_) capable of inhibiting the development of larvae to the adult stage (100% mortality of exposed larvae) was determined as well as the highest concentration (HiC) not having any observable impact on the growth of larvae (0% mortality of exposed larvae). In between both concentrations, the LC_50 _(concentration leading to the mortality of 50 % of exposed larval population) was also determined for each PP.

### Operational importance of the LoC_100 _and the HiC

Operationally, the importance of determining the LoC_100 _resides in the fact that this is the concentration which produces the best lethal larvicidal activity with fewer products. At this concentration, the treatment process is financially economical. As for the HiC, also referred to as the NOEL (no effect level) this corresponds to the quantity of petroleum products "wasted" in the environment during treatment of breeding sites. The HiC is the concentration that has no observable toxicity on *Anopheles *larvae and is, therefore, the concentration at which mutant or resistant populations of *Anopheles *could be gradually selected. Both operational concentrations were the main focuses of this field and laboratory investigations on malaria control tools and the selection of resistance in malaria vectors.

### Laboratory treatment of breeding sites and, identification of HiC and LoC_100 _of tested PP

Increasing quantities of each PP were used to treat surfaces of breeding sites constituted in the laboratory. Known volumes of PP were introduced in 255 cm^2 ^bowls each containing well water and 25 larvae of *Anopheles *Ladji (second to third stage larvae). Larvae were fed throughout the experiment with biscuit and yeast. Each dilution was replicated four times, making a total of 100 larvae tested at each concentration. Similar breeding sites with no traces of petroleum products (controls) were constituted and monitored in tandem.

Mortality rates as well as the number of adults emerging from each breeding site were determined. Data generated were pooled and used for plotting the curve of activity of each PP on the resistant *Anopheles *Ladji strain. Thus, the HiC and the LoC_100 _corresponding to petrol, kerosene, engine oil and waste oil was determined by doses response analysis during this laboratory assessment. In between the HiC and the LoC_100_, the LC_50 _was determined for each PP.

### Identification of the mode of action of PP on populations of *Anopheles *Ladj

Two potential modes of action of petroleum products were screened during this study: i) the killing of larvae by "suffocation" through the oil film/layer produced by PP at the surface of breeding sites, and ii) the direct lethal activity through dissolved particles of PP in breeding sites, referred here as "contact toxicity". Water samples with visible residues of petroleum products were collected in the field and more precisely in areas treated with petroleum products. These samples (crude samples from the field) were used in the laboratory for the simulation of two types of breeding sites:

i) The first set of breeding sites known as "unsieved or crude" was directly reconstituted by putting two litres of the water from the field into laboratory bowls.

ii) The second set of breeding sites known as "sieved or clean" was reconstituted by sieving to clean two litres of the crude sample from the field.

One hundred larvae of *Anopheles *Ladji were introduced in each bowl and reared to adults. Four replicates were made for each simulation, making a total of 400 larvae monitored in "un-sieved/crude" and "sieved/clean" breeding sites. Control bowls were constituted alongside using well water with no traces of petroleum products and containing the same number of *Anopheles *larvae. For each type of breeding site (sieved and un-sieved), mortality rates were determined and statistical tests of comparison used to associate larval lethality with either sieved or un-sieved simulations.

### Data analysis

Sociological information from focus group discussions, in-depth interviews and questionnaires conducted in studied communities were compiled and analysed using Excel and Text base-beta computer software.

Larvicidal activities of each PP on the resistant strains Ladji were analysed and plotted with Excel and, key values tested with Stat-calc-Epi-info software. The potential mode of action of PP on *Anopheles *Ladji was determined through statistical comparison using Stat-calc-Epi-info software.

## Results

### Qualitative and quantitative analysis of sociological information on the empirical utilisation of PP in traditional communities

**Figure 1:**
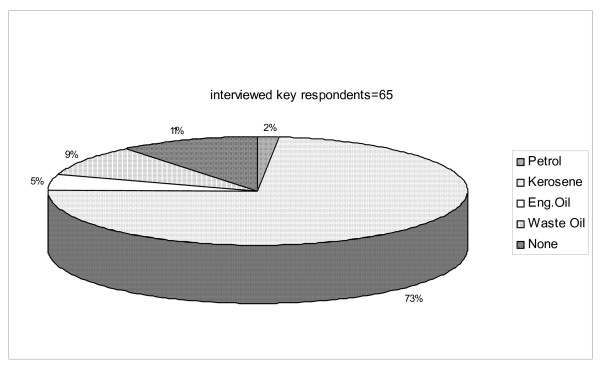
Utilization of PP in three traditional communities in areas of mosquito resistance in the Republic Benin.

Results from qualitative and quantitative surveys conducted in some traditional communities (Gbodjo, Ladji, and Ketonou) in the Republic of Benin and more specifically in localities where *Anopheles *populations are resistant to standard insecticides (mortality rates with permethrin < 60%,) confirmed the empirical use of PP against several insects of great nuisance like: flies, mosquito larvae and cockroaches. The PP mainly used in these communities are kerosene and waste engine oil from mechanics. Others, such as petrol and engine oil, are said to be used occasionally. These products are either sprayed on the ground, on table surfaces, on standing water points and in latrines. Out of a total number of 65 key respondents interviewed in the three communities, 73% spray kerosene in standing water points and latrines to control mosquito growth and nuisance, whereas 9% use waste engine oil, 5% engine oil and 2% petrol. Information extracted from the interviewed populations also showed that 11% do not know much about PP. This sociological investigation revealed that this technique of controlling vectors using PP is transferred in the community from parents to offspring without any scientific explanation to support the efficacy of this strategy (Figure 1).

### Quantification of the lethal activities of PP on the resistant strain *Anopheles *Ladji

#### Lethal activity of petrol

**Figure 2:**
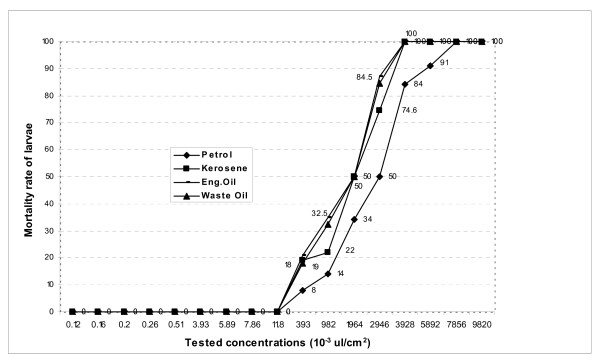
Quantification of the larvicidal activities of PP on *Anopheles *Ladji (Resistant strain).

Experiments conducted in the laboratory on the larvicidal activities of petrol on larvae of *Anopheles *Ladji showed 100% mortality when treatment concentrations of 7,856 × 10^-3^μl/cm^2 ^were reached in experimental bowls. The lowest concentration (LoC_100_) of petrol capable of producing 100% mortality was, therefore, recorded at 7,856 × 10^-3^μl for each cm^2 ^of treated breeding site. As for the highest concentration (HiC) of petrol yielding no larvicidal activity on the resistant strain Ladji, this was recorded as 11.8 × 10^-3^μl for each cm^2 ^of treated surface. LC_50 _obtained for the petrol was 2,946 × 10^-3^μl/cm^2 ^These results imply that any treatment of breeding sites with concentrations below 7,856 × 110^-3^μl/cm^2 ^are likely not to have a lethal effect on the larvae and is, therefore, the threshold concentration below which resistant strains stand high chances of selection (Figure 2).

#### Lethal activity of kerosene

Kerosene was highly effective on *A. gambiae *Ladji at concentrations ranging from 11.8 × 10^-3^μl to 3,930 × 10^-3^μl per cm^2 ^of treated surface. Corresponding mortalities shifted the lethal curve from 0% to 100% (Figure 2). The highest concentration (HiC) of kerosene yielding no larvicidal activity on *Anopheles *Ladji was recorded at 11.8 × 10^-3^μl/cm^2^, whereas the lowest concentration (LoC_100_) producing 100% mortality was recorded at 3,930 × 10^-3^μl/cm^2 ^and the LC_50 _was 1,964 × 10^-3^μl/cm^2 ^(Figure 2).

#### Lethal activity of engine oil

Engine oil was effective on the resistant strain Ladji after treatments of simulated breeding sites with concentrations between 11.8 × 10^-3^μl/cm^2 ^and 3,930 × 10^-3^μl/cm^2^. The HiC and the LoC_100 _recorded with engine oil were respectively 11.8 × 10^-3^μl/cm^2 ^and 3,930 × 10^-3^μl/cm^2^. The LC_50 _was identified at 1,964 × 10^-3^μl/cm^2 ^(Figure 2).

#### Larvicidal activity of waste engine oil

Treatment with waste engine oil exhibited mortalities of 100% at concentrations equal or higher than 3,930 × 10^-3^μl/cm^2^. The highest concentration of waste oil having no lethal activity on larvae of *Anopheles *Ladji was recorded at 11.8 × 10^-3^μl/cm^2 ^and the LC_50 _1,964 × 10^-3^μl/cm^2 ^(Figure 2).

#### Comparative analysis of HiC and LoC_100 _of analysed petroleum products

**Figure 3:**
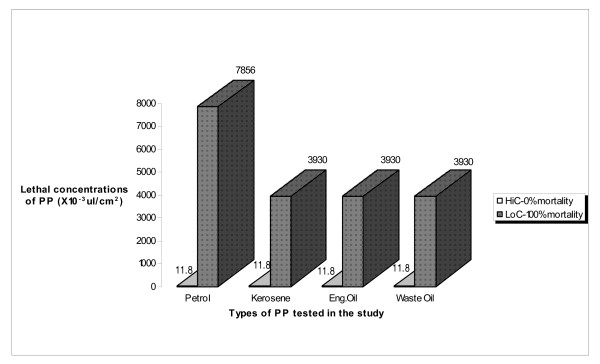
Comparative analysis of the HiC and LoC_100 _of tested PP on larvae of *Anopheles *Ladji (Resistant strain).

Comparative analysis of the lowest concentrations of petrol, kerosene, engine oil and waste oil producing 100% mortality on larvae of *A. gambiae *Ladji showed that kerosene, engine oil and waste engine oil had LoC_100 _of 3,930 × 10^-3^μl/cm^2^, whereas the LoC_100 _recorded with petrol was almost twice as much (7,856 × 10^-3^μl/cm^2^). This comparative analysis highlighted the relatively low larvicidal activity of the petrol on larvae of *A. gambiae *Ladji compared to other PP. As for the HiC, similarities were recorded with all the four petroleum products: HiC = 11.8 × 10^-3^μl/cm^2 ^(Figure 3).

#### Identification of the mode of action of PP on larvae of *Anopheles gambiae Ladji*

**Figure 4:**
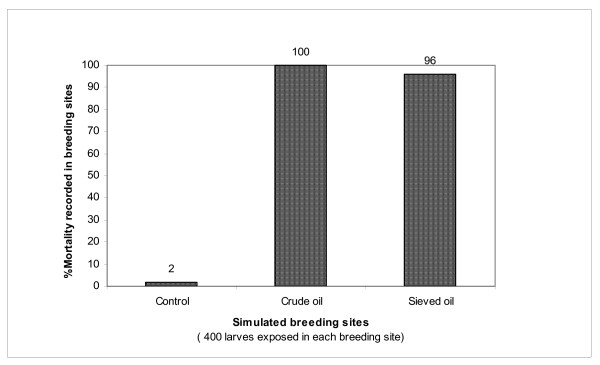
Mode of action of PP on larvae of *Anopheles *Ladji (Mortality rate in unsieved and sieved breeding sites).

The analysis of the mode of action of PP on mosquito larvae showed that 100% (n = 400 exposed larvae) of the larvae were killed in laboratory bowls containing crude petroleum products with oil pellicle (oil film) on the surface. However, when this pellicle was completely removed through a series of sieving, the mortality dropped to only 96% (Figure 4). This insignificant decrease (Pv = 0.000053) in the mortality after the removal of the oil film suggests that 4% of larvae death is associated to the « suffocation » due to the presence of the oil pellicle, whereas the majority (96%) death is associated to the « contact toxicity » with dissolved compounds of PP in the sieved water (Figure 4).

## Discussion and conclusion

### Empirical utilization of PP in traditional communities in Benin

Although abandoned long ago by National Programmes of Malaria Control in most African countries, PP are still used in several traditional communities in the Republic of Benin. This is probably due to the availability of these products, which are sold on the streets of many cities in this country and, are often freely available from mechanical workshops and industries (case of waste engine oil). The low income of communities in traditional settings also accounts immensely in the slow adoption of synthetic insecticides and, therefore, solidifies their attachment to PP as the main malaria control tool. The use of PP for vector control in the studied communities is transferred from generation to generation without any scientific explanation. The availability of PP in several localities in the Republic of Benin and their relative cost-effectiveness suggests this method of mosquito control may have some benefit to the communities.

### Lethal activity of PP on *Anopheles *ladji

This study has clearly established the larvicidal properties of petrol, kerosene, engine oil and waste oil against *Anopheles *Ladji larvae, the most resistant strain currently identified in the Republic of Benin [13]. The relatively low efficacy of petrol as recorded during this experiment could be explained by its high volatility compared to kerosene, engine oil and waste oil. The high volatility of petrol does not allow its persistency in breeding sites. This low persistency results in a low residual effect of this product in treated breeding sites. The relatively high efficacy of kerosene, engine oil and waste oil is likely to be due to their elevated persistency in breeding sites after treatment.

The HiC and the LoC_100 _defined and determined in this research are key operational values for this assessment which focused on the analysis of malaria control tools and the selection of resistance in *A. gambiae*. The importance of the LoC_100 _resides in the fact that this is the concentration which produces the best lethal activity with fewer products. It, therefore, corresponds to the cost of product during the treatment of breeding sites. On the other hand, the HiC corresponds to the quantity of petroleum products "wasted" in the environment during treatments of breeding sites. This value defines the threshold at which resistant populations of *Anopheles *could be gradually selected. The HiC values recorded in this study reflects the high probability of theses products to contribute in the emergence of resistant populations of *Anopheles *in studied localities. It is possible that these products might have contributed through a form of cross-resistance to the elevated cases of pyrethroid resistance currently recorded in various localities in the Republic of Benin. The empirical use of PP and the absence of knowledge on ideal concentrations to use during the treatment of breeding sites have over the years contributed to the spread in the environment of none active quantities of PP which in return would have contributed to the selection of resistant populations of mosquitoes. These 'mosquito-friendly' concentrations of PP found in standing water bodies (HiC) have probably induced over the years a cross resistance to pyrethroids in *Anopheles *populations. However this hypothesis needs to be investigated thoroughly using advanced molecular techniques.

The basic information revealed by this study on the mode of action of PP on mosquito larvae highlights an active lethality of PP by "contact-toxicity" rather than suffocation. It is possible that some heavy metals from petroleum products dissolve, diffuse into the water and are ingested by *Anopheles *larvae after treatment of the breeding sites. The elevated mortality in sieved breeding sites probably occurs following the ingestion of these metals by larvae. In-depth analyses have to be conducted to identify and quantify these metals and to monitor their progression in the digestive system of *Anopheles *larvae after ingestion.

## Conclusion

This study investigated the efficiency of the empirical treatment of *Anopheles gambiae *breeding sites with petroleum products in areas of insecticide resistance in the Republic of Benin. Corroborative results from focus group discussions organized within communities and the laboratory analysis of samples revealed the lethal activity of PP on resistant strains of *Anopheles*. While highlighting the mode of action of PP on *Anopheles *larvae (contact toxicity rather than suffocation), data from this research have also pointed out some non-lethal or "larval-friendly" concentrations which could gradually contribute to the emergence of resistant populations of *Anopheles *in the locality.

Further investigations using advanced molecular techniques and technologies should be envisaged to fully identify the various pathways of petroleum compounds when ingested by *Anopheles *larvae and the contribution of PP in the selection of insecticide resistant populations of *A. gambiae*. Micro-array analyses of samples of *A. gambiae *using detox-chips are currently being conducted in collaboration with the Liverpool School of Tropical Medicine to elucidate the gene expression profile of species of *Anopheles *selected with petroleum products. This advanced analysis of samples will contribute to highlighting the possible implication of petroleum products in the emergence of pyrethroid resistance in some countries of West Africa.

## Authors' contributions

RFD conceived the study and participated in the implementation, data interpretation and manuscript preparation. AAB and HSB contributed in the study design and were fully involved in the implementation of this research and the write up of the manuscript. JMCD contributed in the study design and data analysis. HK participated in the design of the study and substantially helped draft the manuscript. MCA guided the study from the conception to the manuscript finalization.
